# SAPHO-Syndrom: Ein Überblick und nosologische Differenzierung von 35 Krankheitsfällen

**DOI:** 10.1007/s00393-021-00979-4

**Published:** 2021-03-16

**Authors:** Philipp Klemm, Uwe Lange

**Affiliations:** grid.8664.c 0000 0001 2165 8627 Abt. für Rheumatologie, klinische Immunologie, Osteologie und Physikalische Medizin, Kerckhoff-Klinik GmbH, Campus Kerckhoff der Justus-Liebig-Universität Gießen, Benekestr. 2–8, 61231 Bad Nauheim, Deutschland

**Keywords:** Chronisch rekurrierende multifokale Osteomyelitis (CRMO), Spondarthritis hyperostotica pustulo-psoriatica, Sternoklavikuläre Hyperostose, Chronische Osteomyelitis, Anterior-chest-wall-Syndrom, Chronic recurrent multifocal osteomyelitis (CRMO), Spondylarthritis hyperostotica pustulo-psoriatica, Sternoclavicular hyperostosis, Chronic osteomyelitis, Anterior chest wall syndrome

## Abstract

Beim SAPHO-Syndrom handelt es sich nicht um eine Entität, sondern um einen inhomogenen, nosologisch wie pathogenetisch heterogenen Symptomenkomplex. Klinisch imponieren subakute, rezidivierende und/oder chronische Krankheitsprozesse mit charakteristisch gemeinsamer Haut-Knochen-Assoziation („ski[n]bo[ne]-disease“). Die chronisch rekurrierende multifokale Osteomyelitis (CRMO) ist die häufigste „SAPHO“-Erkrankung im Jugend- und Adoleszentenalter. Im Erwachsenenalter ist die Spondarthritis hyperostotica pustulo-psoriatica mit der Trias palmoplantare Pustulose, sternokostoklavikuläre Hyperostose und ossifizierenden Wirbelsäulenmanifestationen am häufigsten und generell als „SAPHO“ bekannt. Zusätzlich gibt es jedoch abortive Krankheitsformen: das entzündliche Anterior-chest-wall-Syndrom, das sternoklavikuläre Hyperostosesyndrom, die Akne-CRMO und die Akne-Spondarthritis. Insgesamt heilen die SAPHO-Krankheitsfälle meist mit relativ günstiger Prognose aus, es gibt aber auch ungünstige Verläufe mit funktionellen Einschränkungen. Neben der typischen Klinik dienen Bildgebung (Röntgen, Szintigraphie, Magnetresonanztomographie) und/oder histologische Knochenbiopsieanalyse der Diagnosestellung. Die Therapie sollte interdisziplinär erfolgen. Eine Antibiose ist obsolet. Der vorliegende Artikel vermittelt einen Überblick über das SAPHO-Syndrom und eine klinisch-rheumatologische wie bildgebende Differenzierung sowie nosologische Klassifizierung von 35 Fällen bei Erstvorstellung.

## Abstract (en)

The SAPHO syndrome is not a single entity but an inhomogeneous, nosologically heterogeneous complex of symptoms with unknown etiology and heterogeneous pathogenesis. Clinically subacute, recurrent or chronic disease processes and a common skin-bone association (skibo disease) can be found. Under the acronym SAPHO, chronically recurrent multifocal osteomyelitis (CRMO) is the most common disease that can occur in youth as well as adolescence. Spondylarthritis hyperostotica pustulo-psoriatica with the triad palmoplantar pustulosis, sternoclavicular hyperostosis and ossifying spinal manifestations is the most common SAPHO form found in adults. Abortive disease forms are the inflammatory anterior chest wall syndrome, extended sternoclavicular hyperostosis syndrome of the clavicle bone, acne CRMO and acne spondylarthritis. The SAPHO disease usually heals with a relatively favorable prognosis but there are also unfavorable courses with functional limitations. The diagnosis should be made based on clinical examination, imaging (x-ray, scintigraphy, magnetic resonance imaging) and/or histological bone biopsy analysis. Treatment should be interdisciplinary. Antibiotic treatment is obsolete. This article provides an overview of the SAPHO syndrome and a clinical-rheumatological imaging differentiation as well as classification of 35 cases at first presentation.

„SAPHO“ ist ein Akronym der Symptome *S*ynovitis, *A*cne pustulosa, *P*ustulose, *H*yperostose und *O*steitis. Das SAPHO-Syndrom wird auch als „Skibo-disease“ („skin and bone disease“) bezeichnet, da eine charakteristische Assoziation pathologischer osteoartikulärer und dermatologischer Symptome vorliegt. Somit ist „SAPHO“ ein Symptomkomplex bzw. ein Syndrom und keine einzelne Krankheitsentität [1–5]. Erstmals wurde das SAPHO-Syndrom von Chamot et al. 1987 beschrieben [6]. Charakteristisch und pathognomonisch für das SAPHO-Syndrom ist eine sterile Osteitis (v. a. im Sinne von Spondylitis und/oder Arthritis) in Assoziation mit Hautmanifestationen, in der Regel im Sinne einer Psoriasis pustulosa, palmoplantaren Pustulose oder einer (pustulösen) Akne. Durch die Verknüpfungsmöglichkeiten der einzelnen Symptome, v. a. jedoch durch die unterschiedliche Ausprägung der „Knochen“- und/oder „Haut“-Symptome ergeben sich erkennbar unterschiedliche Krankheitseinheiten/-entitäten des SAPHO-Syndroms.

## Ätiologie und Prävalenz

Die Ursache für das SAPHO-Syndrom ist nach wie vor unbekannt. Zudem dürfte bei der nosologischen Spannweite der konstituierenden Krankheiten mit keiner einheitlichen Krankheitsursache zu rechnen sein. Bis auf Ausnahmen sind auch genetische Zusammenhänge noch unklar. Für die chronisch rekurrierende multifokale Osteomyelitis (CRMO) als sterile Osteomyelitis sind mehrfach das *Propionibacterium acnes* und koagulasenegative Staphylokokken als mögliche potenzielle antigene Trigger beschrieben [7]. Diskutiert wird, dass die Erreger im Knochenmark eine blande Inflammation auslösen mit immunologischer Prägung und lymphoplasmazellulären Infiltraten, die später mit konsekutiv reaktiv sklerosierenden und hyperostotischen Veränderungen („sklerosierende Osteomyelitis“) einhergehen [8, 9]. Ein familiäres Vorkommen der CRMO ist beschrieben, auch bei Zwillingen [10]. Das SAPHO-Syndrom ist weltweit verbreitet mit einer geschätzten jährlichen Prävalenz von < 1:10.000 in der weißen Bevölkerung und 0,00144 auf 100.000 Japanern [11]. Weltweit sind bisher über 1000 Fälle berichtet worden [12, 13].

## Klinische Manifestationen beim SAPHO-Syndrom

Osteitis und Hyperostose stellen die zentralen Manifestationen des SAPHO-Syndroms dar, die typischerweise mehrere Bereiche betreffen können mit der Folge von irreversiblen Knochen- und Gelenkschäden. Fasst man die Daten der internationalen Literatur zusammen (aktuelle Übersicht in [5]), so ergibt sich folgendes Bild: Am häufigsten betroffen ist die vordere Brustwund (Anterior-chest-wall[ACW]-Syndrom), gefolgt von einem Befall des Achsenskeletts (Wirbelsäule und Iliosakralgelenke), der langen Extremitätenknochen und peripheren Gelenke. Die ACW-Beteiligung findet sich in 65–90 % der Fälle [14–17], typisch dabei betroffene Strukturen sind die Sternokostal- und Sternoklavikulargelenke und das Ligamentum costoclaviculare [15, 16]. Eine axiale Manifestation weisen etwa 32–52 % der Patienten auf [15, 16], einen Befall peripherer Knochen und Gelenke 65–83 % [13, 18]. Eine kutane Beteiligung beim SAPHO Syndrom ist häufig, meist in Form einer palmaren-plantaren Pustulose (ppP) und schweren Akne [19]. In einer monozentrischen chinesischen Kohortenstudie [13] von 354 SAPHO-Betroffenen aus dem Jahr 2019 konnte bei 94 % eine Hautbeteiligung (davon in 92 % eine ppP, die restlichen in Form einer schweren Akne, einer Psoriasis vulgaris und in wenigen Fällen einer psoriatischen Nagelbeteiligung) nachgewiesen werden. In > 70 % entwickelten sich kutane und osteoartikuläre Manifestationen innerhalb von 2 Jahren [13, 20]. An extraartikulären Manifestationen sind entzündliche Darmerkrankungen [21, 22], Lungenbeteiligung [23], Gefäßstenosierung (meist der A. subclavia bei Osteitis subclavia [24]), neurologische Komplikationen (Plexusneuritis bei Spondylitis der unteren zervikale Wirbelsäule, Interkostalneuralgie), Pleuritis und Perikarditis (bei oberer Thoraxosteitis – ACW-Syndrom) [4, 5, 24] sowie Uveitis [25] beschrieben.

Eine umfassende Bewertung ist wichtig für die Diagnose und Behandlung des SAPHO-Syndroms. Je nach vorliegender Krankheitsentität zeigen sich einzigartige klinische Befallsmuster. Das bisher einzig publizierte deutsche SAPHO-Kollektiv [4] stellt sich wie folgt dar:

### Spondarthritis hyperostotica pustulo-psoriatica (SHPP) und Abortivformen

Die SHPP ist landläufig als „SAPHO“ bekannt, nach Schilling [4] jedoch eine der „Reinformen“ des SAPHO-Syndroms. Typischerweise ist die SHPP HLA-B27 negativ und kommt ausschließlich im Erwachsenenalter vor. Die Erkrankung ist durch enthesitische Veränderungen geprägt mit der Trias aus 1. sternokostoklavikulärer Hyperostose mit ossifizierender Periostitis, meist asymmetrisch und evtl. komplikativ durch eine Stenose der V. subclavia, 2. produktiver, hyperostotisch, syndesmophytär oder parasyndesmophytär ossifizierender Spondylopathie und 3. palmarer-plantarer Pustulose oder Psoriasis vulgaris (Typ Königsbeck-Barber). Fakultative Manifestationen von Arthritiden (meist Oligoarthritis) und Sakroiliitis sind möglich [4, 5]. Im Mainzer SAPHO-Kollektiv hatten 25 % diese Manifestationsform.

Wenn die volle Trias der SHPP nicht erfüllt wird, jedoch die klassische sternokostoklavikuläre Hyperostose mit ossifizierender Periostitis vorliegt, sollte an isolierte oder oligosymptomatische Formen der sternokostoklavikulären Hyperostose (SCCH) als Abortiverkrankung zur SHPP nachgedacht werden. Oftmals tritt die SCCH in Kombination mit Symptomen undifferenzierter Spondylarthritiden, mit und ohne pustulöse Dermatose (aber ohne die volle Trias der SHPP!) auf.

### Chronisch rekurrierende multifokale Osteomyelitis (CRMO) und Abortivformen

Die CRMO als die zweite „Reinform“ des SAPHO-Syndroms [4] galt viele Jahre als eine entzündlich benigne Knochenerkrankung des Kindes- und Jugendalters mit weiblicher Dominanz, später wurde auch die adulte CRMO beschrieben (mit Beginn ab dem 20. Lebensjahr) [26]. Dabei wurden verschiedene Formen mit subakuten, rezidivierenden und chronischen Verläufen detektiert sowie diverse Befallsmuster: vordere Thoraxwand, Röhrenknochen, Becken [27], Wirbelsäule [4], Kieferknochen und chronische Arthritis [28]. Als pathognomonisch wird dabei der histopathologische Befund mit lymphoplasmazellulärer Entzündung beschrieben ohne Nachweis von Keimen [4]. In etwa 50 % der Fälle manifestiert sich ein entzündliches Syndrom der vorderen Thoraxwand („anterior chest wall“ [ACW]) als eine Abortivform der CRMO [4]. Beim sog. „Beckentyp“ liegt eine sehr schmerzhafte Koxitis vor [2]. Bei einem Befall der Röhrenknochen im Bereich der unteren Extremität sind die benachbarten Gelenke oft arthritisch mit betroffen [28]. Die adulte Form imponiert mit einem Befall der Femurdiaphysen. Eine chronische Osteomyelitis der Klavikula ist charakteristisch, kommt aber auch solitär vor [29]. An der Wirbelsäule können ein und mehrere Wirbelkörper betroffen sein [30], sekundär ist auch eine Spondylodiszitis beschrieben [31] und ein solitärer Befall der Mandibula [32]. Bei der adulten CRMO kommt es in 70 % zu einem Hautbefall (palmoplantare Pustulose, Psoriasis vulgaris), hingegen nur in bis zu 30 % bei der kindlichen und jugendlichen CRMO, wobei hier auch eine pustulöse Akne vorkommen kann [4, 33]. Der Overlap CRMO und pustulöse Akne stellt dabei eine Abortivform (Akne-CRMO bzw. Akne-Spondarthritis, je nach Befall) dar [4]. Im Labor finden sich neben erhöhter Blutsenkungsgeschwindigkeit (BSG) und erhöhtem C‑reaktivem Protein (CRP) bei fehlender Leukozytose keine richtungsweisenden Befunde [4, 5]. Die Serologie ist bis auf evtl. leicht erhöhte antinukleäre Antikörper(ANA)-Titer unauffällig. In Falldarstellungen ist die Koinzidenz mit einer Takayasu-Arteriitis [34], pulmonalen Erkrankungen [4, 23], Borreliose [8], Morbus Behçet [35] und erythropoetischer Anämie [26] beschrieben.

### Weitere Abortiverkrankungen

Wie beschrieben, stellt das erweiterte ACW-Syndrom eine undifferenzierte CRMO dar [35] und die SCCH eine undifferenzierte SHPP [36]. Liegt ein Hautbefall in Sinne einer Acne conglobata oder Acne fulminans vor, sind die skeletalen Veränderungen unterschiedlich (Akne-Spondarthritis, Akne-CRMO) [37]. Zu erwähnen bleibt die sehr seltene „enteropathische Form“ innerhalb des SAPHO-Syndroms mit Assoziationen zu den enteropathischen Spondylarthritiden. Im Mainzer SAPHO-Kollektiv fanden sich insgesamt 5 Fälle, 4‑mal davon eine Trias aus CRMO, Morbus Crohn und palmoplantarer Pustulose [22].

Zusammenfassend konnten im Mainzer SAPHO-Kollektiv 50 % der Patienten der CRMO und 25 % der Patienten der SHPP zugeordnet werden. Die restlichen 25 % des SAPHO-Kollektivs entsprachen überlappenden Abortivformen der CRMO bzw. der SHPP.

## Diagnose

Der ersten diagnostischen Kriterien wurden 1988 von Benhamou et al. [38] unter Berücksichtigung klinischer Manifestationen und radiologischer Untersuchungen vorgeschlagen. Weitere international häufig verwendete Diagnosekriterien wurden von Khan und Khan 1994 vorgeschlagen [39] und 2003 überarbeitet [40], hauptsächlich basierend auf klinischen Symptomen (Tab. 1).

**Table 1:** 

Benhamou et al. (1988)	Mindestens 1 der folgenden 4 Bedingungen:
	(1) Osteoartikuläre Manifestationen von Acne conglobata, Acne fulminans oder Hidradenitis suppurativa
	(2) Osteoartikuläre Manifestationen von ppP
	(3) Hyperostose (des ACW, der Gliedmaßen oder der Wirbelsäule) mit oder ohne Dermatose
	(4) CRMO mit Beteiligung des axialen oder peripheren Skeletts mit oder ohne Dermatose
Kahn und Kahn (1994)	Mindestens 1 der folgenden 3 Bedingungen:
	(1) Chronisch rezidivierende multifokale sterile und axiale Osteomyelitis mit oder ohne Dermatose
	(2) Akute, subakute oder chronische Arthritis in Verbindung mit ppP, pustulöser Psoriasis oder SA
	(3) Jegliche sterile Osteitis in Verbindung mit ppP, pustulöser Psoriasis oder SA
Kahn (2003)	Mindestens 1 der folgenden 5 Bedingungen:
	(1) Knochen-Gelenk-Beteiligung in Verbindung mit ppP und Psoriasis vulgaris
	(2) Knochen-Gelenk-Beteiligung in Verbindung mit SA
	(3) Isolierte sterile Hyperostose/Osteitis
	(4) CRMO (Kinder)
	(5) Knochen-Gelenk-Beteiligung in Verbindung mit chronischen Darmerkrankungen
	*Ausschluss:* infektiöse Osteitis, tumoröse Knochenveränderungen, nichtentzündliche kondensierende Läsionen des Knochens

*ACW* „anterior chest wall“,* CRMO* chronisch rekurrierende multifokale Osteomyelitis, *ppP* palmare-plantare Pustulose, *SA* schwere Akne, *SAPHO* Synovitis, Akne, Pustulosis, Hyperostosis, Osteitis

Im deutschen Sprachraum finden sich erste Arbeiten zum SAPHO-Syndrom durch Schilling et al. [1, 4]. Die nosologische Analyse nutze dabei v. a. klinische, radiologische und größtenteils histopathologische Befunde. Insgesamt wurden 5 Krankheitsgruppen herausgearbeitet, von denen 2 (I. und III.) umschriebene Entitäten darstellen und die 3 anderen (II, IV, V) Abortivformen der 2 Erstgenannten mit symptomatisch variablem Syndromcharakter sind (Tab. 2). Diese Kriterien wurden jedoch deutschsprachig publiziert, weshalb sie international wenig Beachtung fanden.

**Table 2:** 

Gruppe	Diagnose	Erklärung
I	Spondarthritis hyperostotica pustulo-psoriatica (SHPP)	Obligate Trias aus palmoplantarer Pustulose, sternokostoklavikulärer Hyperostose und einer (hyperostotischen) Spondylarthritis
II	Isolierte oder oligosymptomatische Formen der sternokostoklavikulären Hyperostose (SCCH)	Abortivform zu I. Oftmals in Kombination mit Symptomen undifferenzierter Spondylarthritiden, mit und ohne pustulöse Dermatose, aber ohne die volle Trias der Spondarthritis hyperostotica pustulo-psoriatica
III	Chronisch rekurrierende multifokale Osteomyelitis (CRMO)	Juvenil-adoleszente bzw. adulte Form mit uni-/multifokaler Osteomyelitis. Oftmals mit pustulöser Dermatose
IV	Isolierte oder oligosymptomatische Formen von entzündlichen Zuständen der vorderen Thoraxwand („anterior chest wall [ACW] syndrome“)	Abortivform zu III. Oft mit pustulösen Dermatosen verbunden, welche auch häufiges Symptom der CRMO darstellen. Hier v. a. Unterschied zu III aufgrund der Lokalisation der Osteitis
V	Osteoartikuläre Manifestationen bei pustulöser Akne, auch als Akne-Spondarthritis und Akne-CRMO bezeichnet	Abortivform zu III. Hier liegt der Unterschied in der Akne (gegenüber der fakultativen pustulösen Dermatose bei CRMO)

*SAPHO* Synovitis, Akne, Pustulosis, Hyperostosis, Osteitis

Gemeinsam ist den SAPHO-Syndromen der klinisch meist rezidivierende oder chronische Verlauf ohne Tendenz zur malignen Entartung oder Septikämie [2–5, 41]. Der Verdacht besteht, wenn eine pustulöse Hautveränderung mit Beschwerden am Bewegungssystem vorliegt. Wenn die weitere Diagnostik als Ursache der Schmerzen eine sterile Knochen- oder Gelenkentzündung ergibt, erhärtet sich die Wahrscheinlichkeit der Diagnose, wobei pustulöse Hautveränderungen nicht obligat sind. Wenn man die nosologische Unterschiedlichkeit des SAPHO-Syndroms bedenkt, ist es einleuchtend, dass es nicht „eine“ sichere SAPHO-Diagnose gibt. Man ist also zu weiterer differenzialdiagnostischer Abklärung verpflichtet, und diese beinhaltet neben einer rheumatologischen und dermatologischen Anamnese und Untersuchung bildgebende Diagnostik (Röntgen, Skelettszintigraphie, Magnetresonanztomographie, Computertomographie) und fakultativ die histologische Analyse einer Knochenbiopsie [4, 5]. Bei vorliegender palmarer-plantarer Pustulose (auch anamnestisch oder familiär bei Psoriasis vulgaris) besteht der dringende Verdacht auf eine CRMO, wenn parallel Schmerzen am Brustbein oder dem Becken mit Arthritis der Hüft- und/oder Knie- und/oder Sprunggelenke vorliegen mit Einschränkung der Funktionalität und Belastungsschmerzen der Wirbelsäule. Bei fehlender Hautmanifestation wird dennoch die Wahrscheinlichkeit einer CRMO erhärtet, wenn die Szintigraphie positiv ist und eine septische Manifestation ausgeschlossen wurde [4, 5, 42]. Die sichere Diagnose der CRMO basiert auf dem Nachweis einer sterilen Knochenmarkentzündung, welche zuerst mittels MRT an den Prädilektionsstellen wie Sternum, Klavikula, Metaphysen von Röhrenknochen, Becken, Wirbelkörper, Fersenbein und Unterkiefer detektiert werden kann [28] und letztendlich durch den histopathologisch-sterilen Nachweis (Infiltration mit nichtputriden Entzündungszellen: Lymphozyten, Plasmazellen) bewiesen wird [1–5]. Neben multifokalen sind auch solitäre Knochenläsionen beschrieben [8, 43]. Interessant ist eine neuere Untersuchung, wonach das ^18^F‑FDG-PET-CT charakteristische und vergleichbare Skelettläsionen aufweist wie die Knochenszintigraphie [44]. Eine Gegenüberstellung der bildgebenden Verfahren ist Tab. 3 zu entnehmen.

**Table 3:** 

Technik	Typische Befunde	Vorteile	Nachteile
Röntgen	Irreguläre Knochenmorphologie, kortikale Verdickungen, erhöhte Dichte der Markhöhle	Ökonomisches und etabliertes Werkzeug zur Erkennung von osteoartikulären Veränderungen	Geringe Sensitivität bei der Erkennung von frühen Läsionen
CT	Knochenhyperplasie und Knochenbrückenbildung an der Ansatzstelle des Ligamentum costoclaviculare	Hohe räumliche Auflösung; die Fähigkeit, osteoartikuläre Veränderungen in frühen Stadien zu erkennen	Geringe Sensitivität bei der Erkennung von Weichteilgewebe; ionisierende Strahlung
MRT	Knochenmarködem (niedriges Signal in der T1WI-Sequenz, hohes Signal in der T2WI- und der Kurz-TI-Inversionserholungssequenz und deutliche Anreicherung)	Nachweis aktiver Entzündung in Knochen- und Weichteilgewebe	Geringe Sensitivität bei der Erkennung struktureller Knochenveränderungen
Knochenszintigraphie	„Stierkopf-Zeichen“ (hohe Aufnahme des Sternokostoklavikulargelenks und des Sternumwinkels)	Nachweis von multifokalen osteoartikulären Läsionen zur gleichen Zeit; Erleichterung der Diagnose von Patienten mit nicht klassischem SAPHO	Schwierigkeit, die Aktivität bzw. Genese einzelner Läsionen zu bestimmen; ionisierende Strahlung
PET/CT	Multiple skeletale Läsionen in der ACW-Region oder Wirbelsäule mit geringer bis mäßiger ^18^F‑FDG-Aufnahme und gleichzeitiger Osteolyse und Osteosklerose	Darstellung der Lage und Verteilung der Entzündung; Erleichterung der Differenzialdiagnose von Knochenmetastasen	Limitierte Fähigkeit zur Bestimmung der Krankheitsaktivität; teuer; nicht ubiquitär vorhanden; ionisierende Strahlung

*ACW* „anterior chest wall“, *CT* Computertomographie, *MRT* Magnetresonanztomographie, *PET* Positronenemissionstomographie, *SAPHO* Synovitis, Akne, Pustulosis, Hyperostosis, Osteitis

## Differenzialdiagnosen

Bei der CRMO kommen differenzialdiagnostisch eine akute (bakterielle) Osteomyelitis, Histiozytose, benigne und maligne Knochentumoren (z. B. Ewing-Sarkom), eine rheumatoide Arthritis und eine ankylosierende Spondylitis in Betracht.

Bei Vorliegen einer SHPP sind differenzialdiagnostisch eine (axiale) Psoriasisarthritis, ankylosierende Spondylitis bzw. axiale Spondylarthritis (axSpA) und primär chronische Osteomyelitiden in Erwägung zu ziehen.

## Krankheitsverlauf

Die SHPP, die nur bei Erwachsenen vorkommt, weist meist einen konstant chronischen Verlauf auf, aktive Krankheitsphasen können bis zu Jahrzehnten andauern. Bei der CRMO liegt in der Regel ein immer wiederkehrender Verlauf (rekurrierend/rezidivierend) vor, wobei allerdings auch subakute Verläufe beschrieben sind mit Ausheilung nach dem 1. Schub [4, 5]. Bei den rekurrierenden Verläufen sind beschwerdefreie Intervalle zwischen einigen Monaten bis Jahren bekannt. Interessant sind die Beobachtungen am Mainzer SAPHO-Kollektiv, hier wurden (i) chronische Verläufe mit „undulierendem Verlauf“ beschrieben, (ii) die längste Krankheitsdauer wiesen Patienten mit CRMO-Manifestation in der späten Kindheit auf mit Transition in die adoleszente Phase [1] mit Verläufen zwischen 2,5 bis 20 Jahren [45]. Komplikativ sind schwere funktionelle Beeinträchtigungen beim Beckentyp durch eine Koxitis und epiphysäre Entwicklungsstörungen mit Störungen des Längenwachstums beschrieben [46]. Liegt eine spondylitische Manifestation vor, sollte eine Gefährdung des Rückenmarks bedacht werden [8].

## Therapie

Bis heute existiert keine spezifische Medikation für das SAPHO-Syndrom. Antibiotika haben therapeutisch auch über einen längeren Zeitraum keine durchgreifende Wirkung erbracht. Die symptomatische Medikation beinhaltet unter rheumatologischen Gesichtspunkten eine Antiphlogese mittels nichtsteroidaler Antirheumatika bzw. Coxibe. Prednisolon sollte nur in Ausnahmefällen eingesetzt werden („stoßweise“). Einzelfallbeschreibungen liegen für krankheitsmodifizierende Medikamente wie Azathioprin, Methotrexat und TNF-α-Blocker vor [24, 47–50]. Neuere Daten zeigen eine erste Wirksamkeit für Tofacitinib zur Behandlung einer Nagel- (und Hautbeteiligung) sowie vorläufige Wirknachweise zur Behandlung der ossären-arthritischen Manifestation [51, 52]. Azithromycin wird probatorisch bei Erwachsenen mit SAPHO-Syndrom wegen seiner antiinflammatorischen Wirkung eingesetzt und bei Kindern mit CRMO, wobei eine zusätzliche Gabe von Calcitonin empfohlen wird [24]. Zunehmend findet sich auch der erfolgreiche Einsatz von Bisphosphonaten bei Erwachsenen und Kindern [24, 53, 54].

Aufgrund des Fehlens von kontrollierten Studien kann keine Aussage zur Effektivität einer medikamentösen Therapie getroffen werden.

Empfehlenswert ist bei allen SAPHO-Fällen eine interdisziplinäre Zusammenarbeit zwischen Rheumatologen, Dermatologen und Orthopäden. Ein weiterer Therapiebaustein ist die physikalische Therapie, die individuell differenzialindikativ erfolgen sollte.

## Klassifizierung eines Krankengutes von 35 Fällen

Da die Diagnosekriterien von Khan et al. aus dem Jahr 1994 und 2003 auf der Basis von klinischen Symptomen beruhen, orientierten wir uns bei der nachfolgenden Analyse unseres Krankengutes an der größten deutschen Studie [4], die sowohl klinische, radiologische und histopathologische Gesichtspunkte bei der Differenzierung (Mainzer SAPHO-Kollektiv) beinhaltete. Insgesamt wurden 35 SAPHO-Fälle aus den Jahren 2000 bis 2020 eingeschlossen, welche sich bei Erstmanifestation vorstellten (Tab. 4).

**Table 4:** 

I. Spondarthritis hyperostotica pustulo-psoriatica (SHPP): Vollbild der Trias *n* = 7; inkomplette Trias *n* = 3
II. Sternokostoklavikuläre Hyperostose (SCCH): *n* = 11, inkomplette SHPP: *n* = 1
III. Chronisch rekurrierende multifokale Osteomyelitis (CRMO): *n* = 16, davon mit ACW-Syndrom: *n* = 3
IV. Entzündliches Syndrom der vorderen Thoraxwand (ACW): *n* = 3, klinisch erweiterte Typen und abortive CRMO-Fälle: *n* = 3
V. Osteoartikuläre Manifestationen der pustulösen Akne: *n* = 2

*SAPHO* Synovitis, Akne, Pustulosis, Hyperostosis, Osteitis

### Spondarthritis hyperostotica pustulo-psoriatica (SHPP)

**Figure 1:**
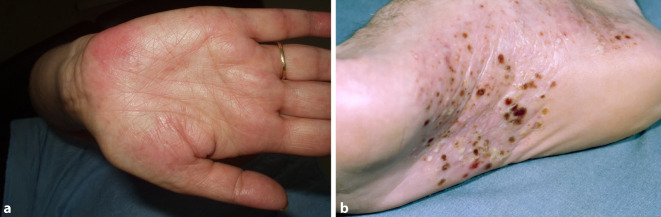

**Figure 2:**
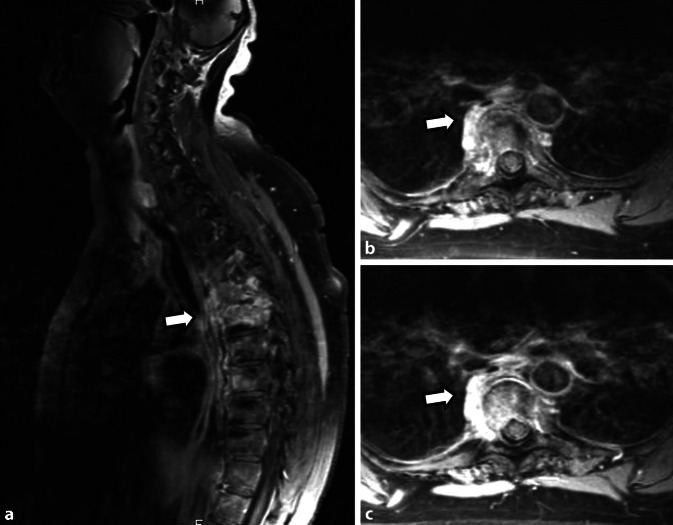

Insgesamt konnten wir 10 erwachsene Fälle diagnostizieren, davon 7 Männer und 3 Frauen. Das durchschnittliche Alter lag bei 32 Jahren (früheste Manifestation mit 18 Jahren, späteste Manifestation mit 56 Jahren). Die Trias (sternokostoklavikuläre Hyperostose – SCCH, Spondylopathie und Dermatose) war 7‑mal komplett und 3‑mal inkomplett gegeben. Eine Dermatose lag in 9 Fällen vor, in 8 Fällen trat sie synchron und in 1 Fall etwa 1 Jahr nach der Skeletterkrankung auf. Bei der Dermatose überwiegt in 8 Fällen die palmare-plantare Pustulose (Abb. 1a, b), davon in 3 Fällen in Assoziation mit einer Psoriasis vulgaris bzw. Onychopathie (Typ Königsbeck-Barber), mit Dominanz der plantaren Pustulose. Aknemanifestationen sind definitionsgemäß nicht berücksichtigt. Der bisherige Verlauf der Erkrankung wurde von den Betroffenen meist als chronisch progredient eingestuft. Die Stammskelettveränderungen (Abb. 2a–c, 3a–c und 4) verteilten sich entsprechend ihrer bildgebungstechnischen Morphologie auf 4 unterschiedliche, z. T. überlappende Typen: Spondylosis hyperostotica 5‑mal, Syndesmophyten 1‑mal, Parasyndesmophyten 1‑mal, Spondylitis 2‑mal, Sakroiliitis 4‑mal (jeweils einseitig). Eine bilaterale Sternoklavikulararthritis konnte 1‑mal mittels MRT (Abb. 5) und eine Gonitis 2‑mal (Arthrosonographie) objektiviert werden. Überwiegend wurden rezidivierende Arthralgien der großen Gelenke angegeben. Eine SCCH war 8‑mal mittels Szintigraphie oder Röntgen diagnostiziert worden, eine durch die SCCH bedingte Subklaviastenose lag in 2 Fällen vor. Schmerzen wurden bei den Patienten entsprechend den entzündlichen Lokalisationen berichtet. Wie auch im Mainzer SAPHO-Kollektiv wurde bei vorliegender Sakroiliitis weder aktuell noch anamnestisch ein entzündlicher tiefsitzender Rückenschmerz angegeben. HLA-B27 war in keinem Fall nachweisbar, CRP und BSG waren z. T. nicht bis allenfalls mäßig erhöht. Histologisch wurde bei den vorliegenden 8 Klavikulaprobebiopsien eine „hyperostotische Spongiosasklerose“ beschrieben.

**Figure 3:**
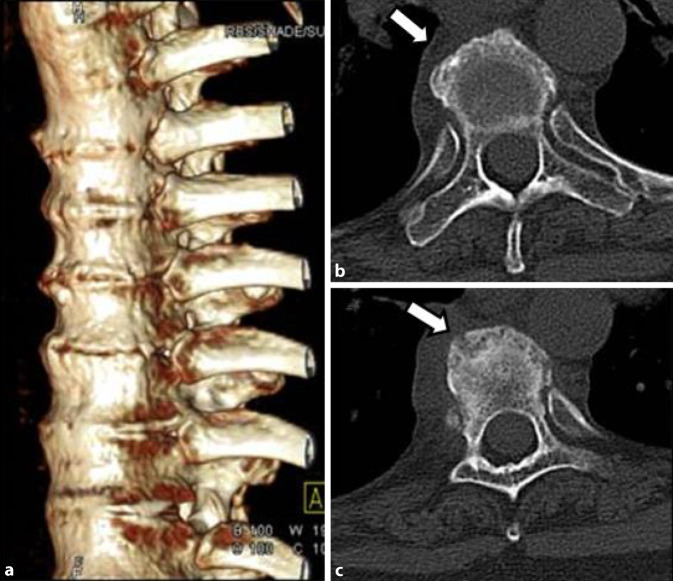

**Figure 4:**
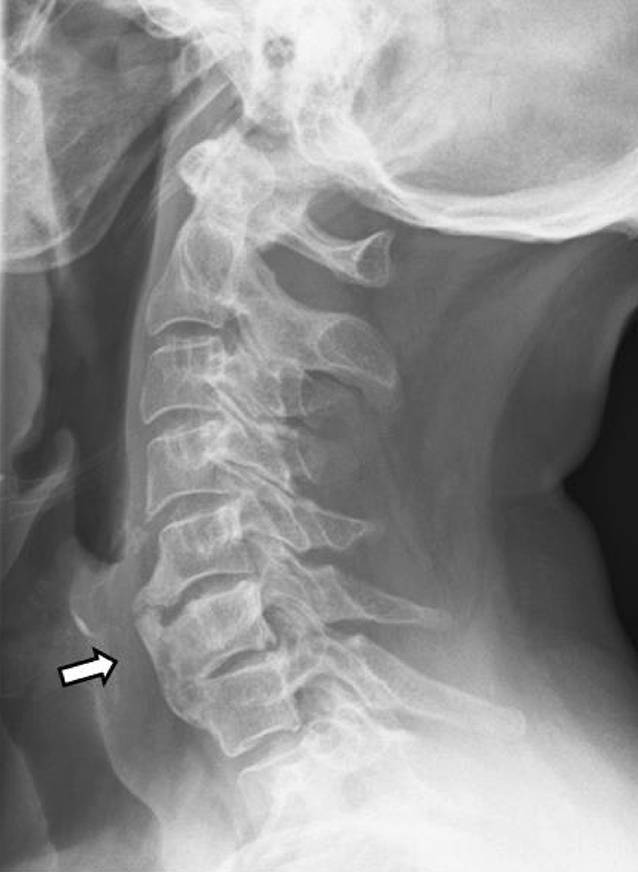

**Figure 5:**
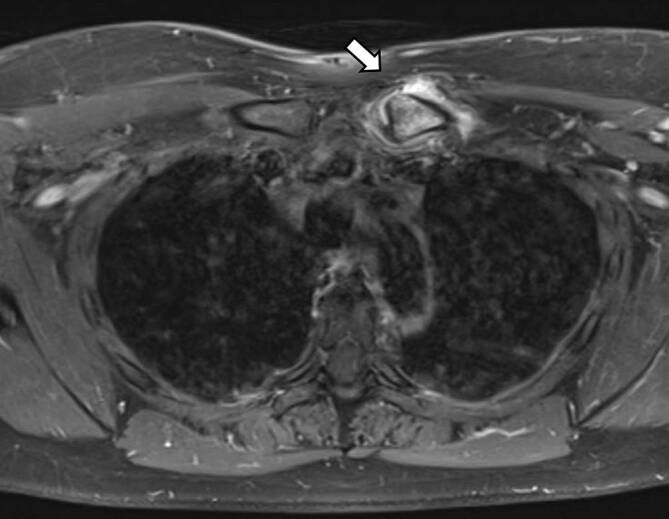

Für den Praxisalltag ist von Bedeutung, dass mögliche Fehldiagnosen wie eine ankylosierende Spondylitis oder Spondylitis psoriatica nur dann gestellt werden, wenn eine Dermatose oder sternoklavikuläre Veränderungen übersehen werden.

### Sternokostoklavikuläre Hyperostose (SCCH) und ihre klinisch erweiterten Syndromtypen

Die SCCH wurde im gesamten Kollektiv 11-mal festgestellt und radiologisch sowie szintigraphisch gesichert. Definitionsgemäß kommt die SCCH, wie unter Tab. 3 dargestellt, gemeinsam bei den Krankheitsbildern I (SHPP) und II (SCCH) vor. Acht von den 11 Fällen einer SCCH konnten der SHPP zugeordnet werden, sodass noch 3 Fälle übrig blieben. Davon boten 2 Fälle (jeweils 1 Frau und 1 Mann) eine isolierte SCCH ohne Dermatose und Skelettbeteiligung. Ein Fall bot eine SCCH mit palmoplantarer Pustulosis (ppP).

### Chronisch rekurrierende multifokale Osteomyelitis (CRMO)

**Figure 6:**
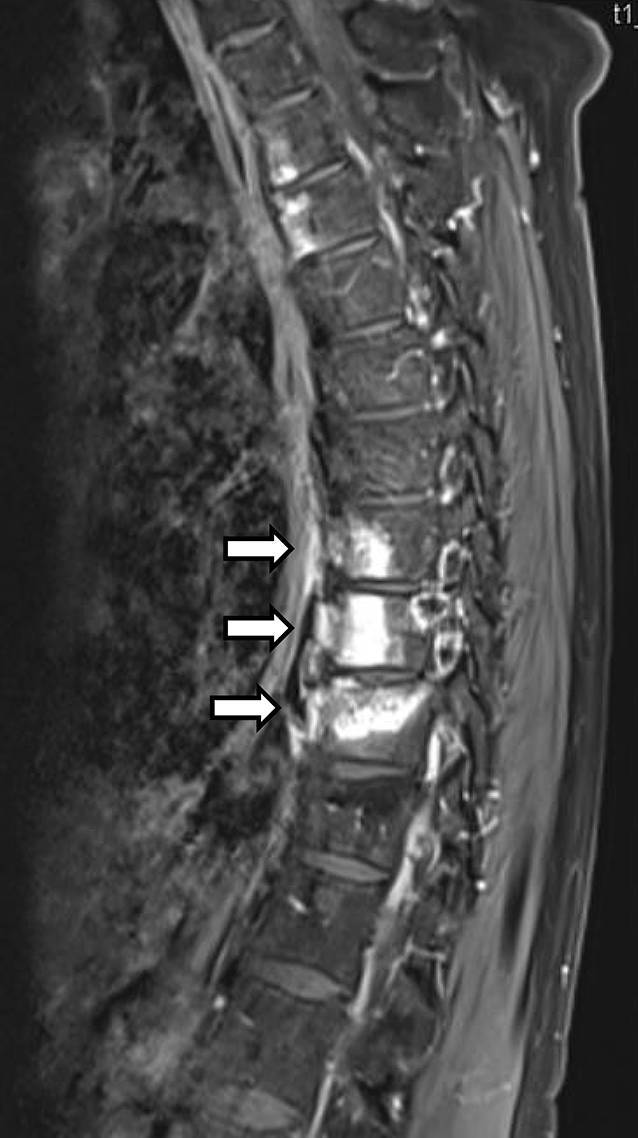

**Figure 7:**
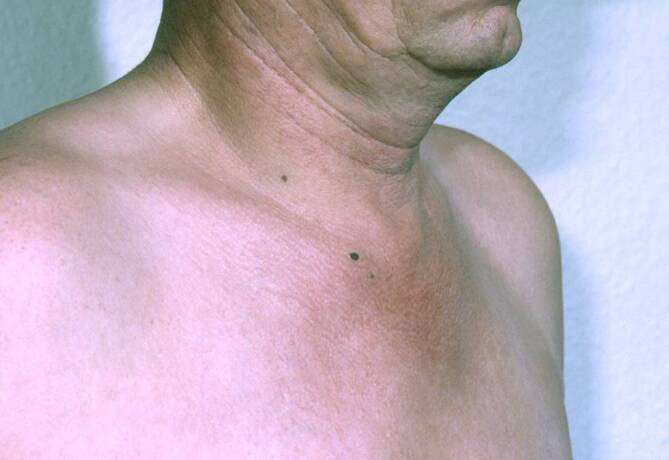

Es konnten insgesamt 16 Fälle (14 adulte, 2 adoleszente) diagnostiziert werden, davon 10 Frauen und 6 Männern, das durchschnittliche Lebensalter zu Krankheitsbeginn lag bei 26 Jahren. Eine psoriatische Dermatose war 13-mal gegeben, davon alle mit einer ppP. Eine dermatoskeletale Assoziation zeigte sich somit in 81,3 %. Zur Orientierung des Skelettbefalls erfolgte eine Szintigraphie, gefolgt von Röntgen und/oder MRT. Der Skelettbefall gliederte sich wie folgt: Spondylopathie der HWS 2‑mal, der BWS/LWS 10-mal (Abb. 6), meta- und diaphysäre Hyperostosen 5‑mal (2-mal Femur, 3‑mal Tibia), 4‑mal einseitige pelvine Hyperostose, 2‑mal Gonitis (in osteomyelitischer Nachbarschaft), knöchern durchbaute Iliosakralgelenke 2‑mal (jeweils einseitig) und 3‑mal ACW-Syndrom (Sternoklavikulararthritis 3‑mal) sowie 1‑mal isolierter Befall der Klavikula (Abb. 7). Im Vergleich zum Mainzer SAPHO-Kollektiv konnten wir keine CRMO der Mandibula diagnostizieren. In den 2 juvenil-adoleszenten Fällen (eineiige männliche Zwillinge) manifestierte sich die CRMO im Bereich der Tibia (1-mal rechts und 1‑mal links). Von den Patienten wurde der bisherige Verlauf als rekurrierend chronisch eingestuft. Bei 14 Fällen ergab die Knochenbiopsie (4-mal Becken, 3‑mal Tibia, 1‑mal Femur, 4‑mal Sternoklavikularbereich, 2‑mal Wirbelkörper) Plasma-sklerotische Prozesse einer chronischen Osteomyelitis. Ähnlich wie beim Mainzer SAPHO-Kollektiv konnten auch wir bei mehreren Fällen eine unnötige operative Intervention nach Diagnosestellung verhindern.

### Entzündliches Syndrom der vorderen Thoraxwand („anterior chest wall“ [ACW]), klinisch erweiterte Typen und abortive CRMO-Fälle

**Figure 8:**
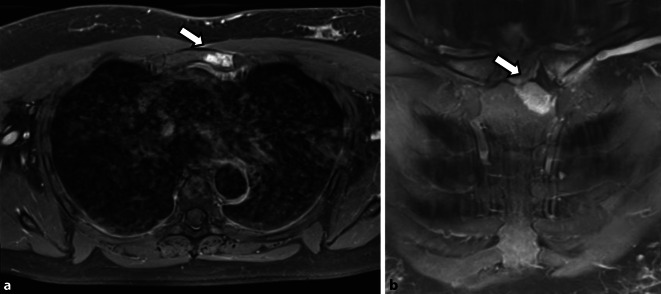

Das entzündlichen Syndrom der vorderen Thoraxwand (ACW-Syndrom) ist gekennzeichnet durch blande entzündliche, synovitische, erosiv-sklerotische und ossifizierende Veränderungen der Sternoklavikularregion im Rahmen einer Sternoklavikulararthritis, Synchondritis manubriosternalis und einer Osteitis des Manubrium sterni (Abb. 8a, b und 9). Klinisch empfiehlt sich, das ACW-Syndrom von den ossifizierenden und hyperostotischen Veränderungen der sternokostoklavikulären Hyperostose abzutrennen, obwohl sich die Symptomatik in Form einer Sternoklavikulararthritis oft überschneidet. Hilfreich ist bei der Unterscheidung die Szintigraphie mit dem hierfür typischen szintigraphischen „Stierkopfzeichen“ beim ACW-Syndrom.

**Figure 9:**
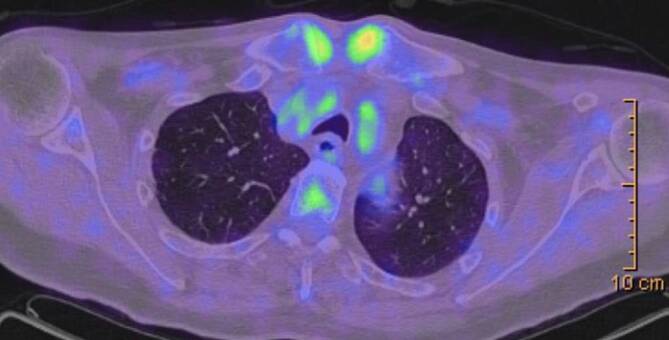

**Figure 10:**
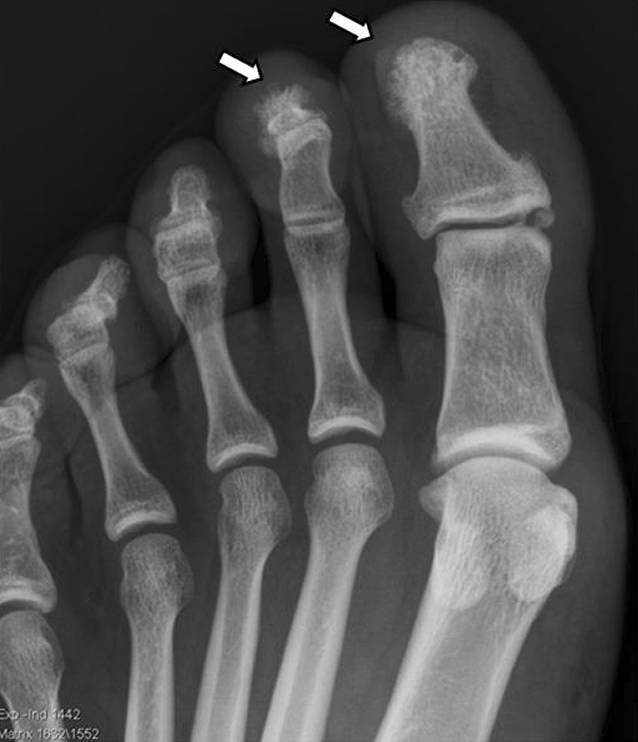

Wir konnten insgesamt 3‑mal ein isoliertes ACW-Syndrom (Sternoklavikulararthritis, Synchondritis sternalis und Sternalsklerose) ohne begleitende Dermatose, Arthritis oder Spondylopathie diagnostizieren, in 1 Fall lag ein ACW-Syndrom mit peripherem arthritischen Befall und einer Psoriasis vulgaris vor (Abb. 10). Bekannt ist, dass diese klinische Manifestation eher selten ist und folgende Typen vorliegen können: ACW-Syndrom mit pustulöser Dermatose (ACW bei ppP oder Akne), ACW-Syndrom mit Oligoarthritis pustulosa (ACW mit ppP oder bei Akne), ACW-Syndrom mit Spondarthritis pustulo-psoriatica und das isolierte ACW-Syndrom.

### Osteoartikuläre (CRMO) Manifestationen der pustulösen Akne

Viele Patienten mit ausgeprägter Akne klagen über Arthralgien, und es kann zu chronisch entzündlichen osteoartikulären Manifestationen kommen. Dabei untergliedert man in (i) die Akne-CRMO, meist mit symmetrischen Befall der Klavikula, histologisch entspricht der Befund einer CRMO; (ii) Spondarthritis hyperostotica acne-pustulotica, meist im frühen Erwachsenenalter bei Vorliegen einer Acne conglobata, häufiger bei Männern und (iii) oligotope Akne-Arthropathie mit der Assoziation Oligoarthritis und Zeichen des ACW-Syndroms bei Acne conglobata oder Acne vulgaris. Meist sind Männer betroffen. In unserem Kollektiv fanden sich 2 Fälle einer Spondarthritis hyperostotica acne-pustulotica.

## Fazit

- Das SAPHO-Syndrom sollte im individuellen Fall nicht als finale Diagnose gesetzt werden, sondern als klinischer „Wegweiser“ dienen.
- Osteitis und Hyperostose sind die zentralen Manifestationen, die kutane Beteiligung kann in Form einer palmaren-plantaren Pustulose, schweren Akne oder Psoriasis vulgaris imponieren.
- Bei Vorliegen einer SAPHO-Symptomenkonstellation sollte eine klinisch-bildgebungstechnische und histopathologisch-rheumatologische Differenzierung erfolgen.
- Die medikamentöse Therapie ist symptomatisch ausgerichtet, eine Antibiose obsolet. Fallberichte über einen Wirksamkeitsnachweis existieren zu Azathioprin und Methotrexat, TNF-Inhibitoren, Tofacitinib, Bisphosphonate, Calcitonin und Azithromycin (aufgrund der antiphlogistischen und immunmodulatorischen Wirkungen).
- Die Therapie sollte interdisziplinär erfolgen und durch physikalisch-therapeutische Maßnahmen individuell bei Dermatosen und Befall des Bewegungssystems ergänzt werden.
